# A qualitative study on safety perception among healthcare workers of a tertiary academic care center during the SARS-CoV-2 pandemic

**DOI:** 10.1186/s13756-022-01068-0

**Published:** 2022-02-08

**Authors:** Selina Ehrenzeller, Ana Durovic, Richard Kuehl, Aurélien Emmanuel Martinez, Michèle Bielser, Manuel Battegay, Matthias von Rotz, André Fringer, Sarah Tschudin-Sutter

**Affiliations:** 1 Division of Infectious Diseases & Hospital Epidemiology, University Hospital Basel, University of Basel, Petersgraben 4, 4031 Basel, Switzerland; 2 University of Applied Sciences and Arts Northwestern Switzerland FHNW, Olten, Switzerland; 3 Institute of Nursing, School of Health Professions, Zürich University of Applied Sciences ZHAW, Winterthur, Switzerland; 4 Department of Clinical Research, University Hospital Basel, University of Basel, Basel, Switzerland

**Keywords:** SARS-CoV-2, COVID-19, Infection prevention and control, Personal protective equipment, Safety, Qualitative study

## Abstract

**Background:**

Protecting healthcare workers (HCWs) from exposure to SARS-CoV-2 during patient care is central to managing the current pandemic. Higher levels of trust in personal protective equipment (PPE) and infection prevention and control (IPC) strategies have been previously related to lower levels of emotional exhaustion, yet little is known on how to achieve such a perception of safety. We thus sought to identify institutional actions, strategies and policies related to HCWs’ safety perception during the early phase of the pandemic at a tertiary care center in Switzerland by interviewing HCWs from different clinics, professions, and positions.

**Methods:**

For this qualitative study, 36 face-to-face semi-structured interviews were performed. Interviews were based on a guide that addressed the perception of institutional strategies and policies during the first phase of the pandemic in March 2020. The participants included doctors (n = 19) and nurses (n = 17) in senior and non-senior positions from eight clinics in the University Hospital Basel, Switzerland, all involved in patient care. All interviews were audio-recorded and transcribed verbatim. Data were analyzed using qualitative content analysis and organized using MAXQDA (VERBI Software GmbH, Berlin).

**Findings:**

Five recurring themes were identified to affect HCWs’ perception of their safety during the SARS-CoV-2 pandemic: (1) transparency and clarity of information, (2) communication on the availability of PPE (with the provision of information alone increasing the feeling of safety even if supplies of PPE were reported as low), (3) uniformity and consistency of guidelines, (4) digital resources to support face-to-face teaching (although personal information transfer is still being considered superior in terms of strengthening safety perception) and (5) support and appreciation for the work performed.

**Conclusions:**

This study identifies institutional policies and actions influencing HCWs’ safety perception during the first wave of the COVID-19 pandemic, the most important of which is the factor of transparent communication. This knowledge reveals potential areas of action critical to improving preparedness and management in hospitals faced with an infectious disease threat.

**Supplementary Information:**

The online version contains supplementary material available at 10.1186/s13756-022-01068-0.

## Introduction

Occupational exposure to SARS-CoV-2 has caused many infections and related deaths among healthcare workers (HCWs) worldwide [1]. Thus, protecting HCWs from exposure to SARS-CoV-2 during patient care has been central to managing the current pandemic. Consequently, institutions strived to secure adequate personal protective equipment (PPE) and to provide infection prevention and control (IPC) guidance. Yet, as knowledge on and training in IPC practices may be limited, such efforts may not suffice to address the needs of frontline HCWs who have experienced substantial emotional and physical stress, especially during the early phases of the pandemic [2–4]. Higher levels of trust in PPE and IPC strategies have been previously related to lower levels of emotional exhaustion [5], yet little is known on how to achieve such a perception of safety and which policies and actions may cause HCWs to feel unsafe. Prior studies conducted on this topic have included HCWs in fairly homogeneous groups of participants in terms of profession, position or department [2, 3, 6, 7]. To gain a broader understanding, we sought to identify institutional actions, strategies and policies related to HCWs’ safety perception during the early phase of the pandemic at a tertiary care center in Switzerland by interviewing HCWs from different departments, professions and positions. We chose a qualitative study approach as it allows a deeper understanding of the HCWs’ experiences with the aim to provide future guidance on how to strengthen HCWs’ safety perception and an institution’s ability to deal with a novel infectious disease threat.

## Methods

### Study design

We used a qualitative descriptive research design [8, 9] that involved conducting semi-structured in-depth interviews with HCWs from key departments involved in the pandemic response at our institutions and deductive-inductive content analysis. Participants were chosen as detailed below. As a quality assessment project, the responsible ethics committee confirmed that approval was not required (EKNZ-Request-2020-00931). Nevertheless, all HCWs provided written informed consent and knew that they could discontinue the interview at any point without negative consequences.

### Setting

The University Hospital Basel is a tertiary care center admitting more than 35,000 adult patients annually and comprising 813 beds. It provides acute care and hospital services in the city of Basel (approximate population: 200,000) and serves as a referral center for patients requiring specialized medical care for the north-western part of Switzerland. At the beginning of the pandemic, a task force was set up to determine the pandemic management at the hospital. The task force, which was chaired by the hospital management and included representatives from various departments, met one to three times weekly.

### Participants

The sampling was purposeful and criterion-based to include HCWs that were involved in direct patient care during the initial acute phase of the SARS-CoV-2 pandemic in March 2020. Employees from different departments that were involved in patient care were eligible for inclusion.

We used email to contact HCWs (n = 36) from eight different clinics, including the medical outpatient clinic, the emergency department, the department of obstetrics and gynecology, the department of anesthesiology, the division of infectious diseases & hospital epidemiology, the intensive care unit, the SARS-CoV-2 cohort ward of the division of internal medicine and the clinic for transplantation immunology and nephrology. We chose these clinics given their key involvement in the management of suspected or confirmed SARS-CoV-2 infected patients. A more detailed description of the characteristics of participants is provided in the Additional file 1: Table S1.

**Table 1:** Main  themes and sub themes relating to safety perception identified during the interviews and mentioned by >85% of participants

**Transparency and clarity of information**
Achieving the feeling of being informed
Transparency of in-house communication
The role of the division of infectious diseases & hospital epidemiology
**Material availability**
Perception of material shortage
Alternative PPE and recycling of masks
**Uniformity and consistency of guidelines**
**Digital resources to support face-to-face teaching**
**Support and appreciation of personnel**

### Data collection

A medical doctoral candidate (SE) conducted face-to-face semi-structured interviews during August and September 2020. She had not been involved in developing the measures taken in the hospital as she started working in the division of infectious diseases & hospital epidemiology after the first wave. She explained the study goal and procedure for the interviews to the participants when obtaining the informed consent and reviewed them again in her opening remarks before the interview.

We developed a semi-structured interview guide (see Additional file 2: Table S2) based on the objectives and the personal experiences with the COVID-19 situation from the perspective of the division of infectious diseases & hospital epidemiology.

The interview guide consists of nine topics which were discussed in a peer debriefing to have been the most important and which were used as a coding frame for data analysis: (1) material availability, (2) IPC guidelines, (3) training opportunities, (4) security (including visitor management), (5) communication, (6) diagnostics and testing strategy, (7) data management, (8) research and (9) preparedness and personal lessons learnt. We digitally recorded and transcribed verbatim all interviews. We excluded personal identifiers.

### Data analysis

We coded the data with deductive-inductive qualitative content analysis according to Schreier [10] to categorize the codes into themes and sub-themes.

We followed a clear step-by-step approach. We categorized the transcripts using a code system based on the main themes deductively taken from the interview guide. In a second step we added subcategories that emerged inductively from the text. During the coding process, we realized that some categories in connection with feelings of insecurity of the HCWs stood out. Consequently, we recoded the entire dataset looking only at “security factors” the HCWs mentioned, which creates a new inductive code system. The subsequent analysis of the individual categories was category-based and comparative between the groups e.g. senior and non-senior employees as well as nurses and physicians.

To ensure the accuracy of the methodology, we involved an external consultant to present the process of coding and methodology that we used and receive feedback on it. We used the consolidated criteria for qualitative research (see Additional file 3: Table S3) to verify the reporting’s completeness [11]. All data were managed and analyzed with MAXQDA 2020 (VERBI Software GmbH, Berlin) [12]. Following the analysis, we carried out member checking with some HCWs, who were asked to give their feedback on the findings (themes and subthemes) to make sure that the findings corresponded with their perceptions [13, 14].

Quotations illustrating the themes were translated from German to English and proofread by two people who made minimal adjustments so as not to lose the meaning of the quotation. The quotations are followed by the interview number, e.g., "P3" for "Physician 3" or "N7" for "Nurse 7". The allocation to position and department can be seen in Additional file 1: Table S1.

It is important to note that numbers have been deliberately omitted and descriptive words such as "most, some, few" are being used instead. The usage of precise numbers and percentages may be misleading and does not add much value to a qualitative study [15, 16].

### Quality criteria

Quality criteria in qualitative research can be summarized as “trustworthiness” [17]. Having achieved trustworthiness means that the study was conducted fairly and according to ethical principles and that the findings represent the experiences of the HCWs as closely as possible [18]. Of the various ways to strengthen the trustworthiness of a qualitative study [16], we chose to use synthesized member checking [19] and peer debriefing with specialists in medicine and qualitative research. We also maintained an audit trail during the coding and analysis process and we are presenting disconfirming evidence.

## Findings

Thirty-six face-to-face semi-structured interviews were performed, which on average lasted for 30 min (range 15–50 min) and resulted in 250 pages of transcript.

Twenty-two (61%) of the participants mentioned that they personally felt safe during work. All HCWs reported difficulties or feelings of insecurity at work. We identified five main themes regarding HCWs’ safety perception. All HCWs discussed “transparency and clarity of information” and “availability of material” and 97% mentioned “uniformity and consistency of guidelines”. Themes and subthemes relating to safety perception, identified during the interviews, and mentioned by > 85% of HCWs are summarized in the Table 1.

### Transparency and clarity of information

#### Achieving the feeling of being informed

Communication and provision of information by hospital management, IPC specialists, and their direct supervisors was essential for employees directly involved in patient care. The HCWs agreed that clear and precise communication was important, especially in the initial phase of a pandemic. The feeling that they were well informed directly induced a sense of security in employees as reflected by the two following statements: *“I think information transfer is a very big safety gain or can create a feeling of safety.” (P1)* and *“The rules of the game with the senior doctors were: "I'll give you the information, but I don't want any complaints if I say the opposite 48 h later". The situation is in flux, and you have to be able to correct misinformation.” (P8).*

The feeling of being well informed differed between clinics and depended on the communication strategy of the respective chief physician. HCWs who had personal contact with members of the task force members or IPC specialists felt better informed than did other HCWs who had no personal contact.

Two employees perceived communication during the first phase of the pandemic as an "information flood", but this perception was not related to a feeling of insecurity.

#### Transparency of in-house communication

Meeting protocols of the task force assemblies and information on PPE availability were not shared with all employees. This lack of information affected the employees’ sense of security as illustrated by the following quotation: “*The [task force decisions] were not always communicated to the outside and it was a bit of a black box. It was "the big task force" that decides something, but it was not always clear what was decided.” (P1).*

A well-placed SARS-CoV-2 information portal on the hospital internet provided a good platform for distributing specific information. HCWs appreciated the information portal since information and directives from hospital management and IPC guidance were well organized and could be accessed easily. A list provided an overview of the current research of important studies.

Employees appreciated being able to access information such as the number of patients presenting to the test center of the hospital, and the current number of hospitalized patients with COVID-19 on the hospital internet information portal. For most HCWs, the information, which was updated regularly, satisfied their need for information and the transparent communication strengthened their trust in the hospital management as well as their sense of security in their work environment. *“This way, without having to violate patient safety or doctor-patient confidentiality, you can have an overview of how things are going in our hospital at any given moment: How many [patients] are in intensive care? How many [patients] are hospitalized?” (P13).*

#### The role of the division of infectious diseases & hospital epidemiology

The division of infectious diseases & hospital epidemiology played a crucial role in offering information to all clinics within the hospital. It provided IPC guidelines that were considered an important source of information and were regularly updated. While HCWs appreciated this openly accessible document, they found it difficult to find specific answers given its size—50 pages—and the supplementary documents. As a result, several employees suggested shorter summaries concerning their specific professions and daily tasks.

HCWs considered the direct exchange with IPC specialists by telephone or regular ward-meetings as very helpful to clarify questions regarding IPC measures and often mentioned this in connection with a resulting sense of security. Some clinics mentioned the need for more support in preparing specific guidelines for their specialized clinics. “*I observed, my colleagues (…) who did not have such an IPC meeting, it was more difficult for them to get information. (…) If I had not had this IPC meeting, it would have been much more difficult for me.” (N6)* The members of the division of infectious diseases & hospital epidemiology found it difficult to keep up to date with changing decisions on IPC, which challenged the division’s ability to provide timely information to all members of the division itself and employees in other disciplines and clinics.

### Material availability

#### Perception of material shortage

Some HCWs perceived that PPE (mainly masks) was in short supply, but others did not. In fact, their perception of PPE availability and their definitions of a shortage varied greatly. Some HCWs perceived a "shortage" due to switching to alternative products: *“In the end, it wasn't a “shortage”, you just had to switch to other companies and other brands, but we actually had enough.” (P4)* For others, the availability of any PPE was proof that there was no shortage in the institution.

Many HCWs felt insecure because they were uncertain about PPE availability, and if so, how serious it was. This feeling of uncertainty was intensified by three factors: (1) the instructions regarding the protective gear changed frequently, (2) alternative PPE was offered and (3) the materials were stored in locked cabinets. The latter was considered necessary because people stole or hoarded masks.

#### Alternative PPE and recycling of masks

Due to global shortages of mask supplies, the hospital procured masks from alternative producers. HCWs found it challenging to use the alternative products because they did not fit well, were of poor quality, or were not familiar. HCWs were uncertain regarding usage of the new PPE and feared being exposed to SARS-CoV-2 during patient care. Many employees wanted clearer communication and instructions regarding the use of the different components of unfamiliar PPE.

Compared with employees in non-senior positions, senior doctors were less likely to perceive alternative products as a severe problem: *“Using alternative products of PPE (i.e. different models or brands) was usually not a problem. Whether the apron is yellow or white, or whether the mask comes from China or Germany, it doesn't matter.” (P8)* However, this statement contrasts with the concerns of non-senior employees, who were very suspicious of the alternative products and were disturbed by "foreign" masks.* “The feeling arose that the hospital does not have enough [supplies of PPE] and that we therefore get second-rate products.” (N8)*

Our institution introduced a policy of collecting used masks for potential recycling in case of further shortages. This policy proved challenging for almost all employees and only a few felt reassured by this measure. Given their (1) lack of familiarity with mask reuse, (2) uncertainty as to whether this was allowed by the manufacturer and 3) fear that the situation was now serious because even used surgical masks were collected. *“In the end, masks were never recycled, we never had to wear recycled masks ever. But it has always been like a sword of Damocles hanging over us and we always thought: “Oh dear, now we'll have to wear recycled masks”, and nobody would have felt safe. And there were quite a few in the team who said, this would be my last day in this hospital when that happens.” (N17)*

### Uniformity and consistency of guidelines

HCWs frequently mentioned the lack of clarity regarding the appropriate use of PPE and uncertainty due to the changing instructions. Non-senior employees mentioned these concerns more frequently than senior employees but without differences between the HCWs from different clinics.

Instructions regarding PPE use were provided and regularly updated to correspond to the current science. While these updates were perceived positively, frequent changes led to confusion and uncertainty. *“At first it was said: "Yes, these masks are useless", then the "FFP masks are better", now I think it is said: "the FFP masks are not really useful either, rather these and…". Just to follow a clear line and clarity: "It's really like this: these masks protect, these don't, these are better." (P13)* When asked personally by employees, some IPC specialists sometimes gave answers contradicting the published guidelines, which added to the HCWs’ sense of insecurity.

Many employees had the impression that the hospital’s and the national recommendations for adequate PPE were adapted to the available material, which fueled HCWs’ sense of uncertainty and increased their distrust in the PPE and the hospital’s pandemic management. *“Somehow you had the feeling that even if you don't have these surgical masks anymore, at some point they'll start saying: "You can also sew it yourself, that's enough." That's just the impression we had.” (N17)* Overall, however, the employees were very aware of the rapidly changing situation and recognized that the division of infectious diseases & hospital epidemiology was working to create the safest possible policies and procedures and aiming to have consistency across the entire institution.

Changes in guidance were usually published on Friday afternoon and this *“led to a lot of confusion, because some already had the new concept, others still had the old one and I think you just have to reckon with that, a hospital is very slow.” (P7)* HCWs perceived the color-coding of the adjustments and short summaries of the changes to be very helpful because they helped HCWs identifying changes.

### Digital resources to support face-to-face teaching

The division of infectious diseases & hospital epidemiology organized training sessions on the wards and provided support by telephone and on-site in case of uncertainties. *“Individuals did a lot of 7-day shifts to instruct the employees and actually it worked out because of individuals, because of personal contacts and not necessarily because there was already a system in place to rely on.” (P19)* However, the great demand for training, especially at the beginning of the pandemic, could not be met with face-to-face training, and digital resources therefore played a major role. *“If you want to train on-site, you need a whole month to do so—that's not realistic. Therefore: video, podcast, whatever. Anything modern is good.” (P8)* In-person training for people who worked only night or late shifts was difficult, as the training usually took place during the day. For these employees in particular, but not exclusively, online training was an important resource. Most of the interviewed staff found online pictorial instructions and videos on simple tasks such as donning and doffing PPE and taking swabs to be as extremely helpful. The videos standardized the procedures, which reinforced the feeling of safety and correct execution. Especially when senior employees in departments had to train non-senior employees themselves instead of receiving training from IPC specialists, they benefited from the online resources as a reference. HCWs’ statements indicated that online training resources should not be seen as an alternative training method but as an addition to on-site training if the resources for the latter are available. *“I think the personal contact has always been well received. But I think that when there is a lot of demand, you can't cover that anymore, so I just think the videos are good, that they are available at all times and that you maybe do additional personal training for important aspects.” (P3)* Additionally, several HCWs mentioned that doctors’ training was not as thorough as that for nurses, even those from the division of infectious diseases & hospital epidemiology itself were less prepared than nurses: *“We think: “We are the specialists”, but in fact, we may not be able to do it so well ourselves.” (P2)* Several HCWs mentioned compulsory IPC training, which would be completed online every 2 years.

### Support and appreciation of personnel

During the acute phase of the pandemic, employees needed support from their direct superiors, department heads, and hospital management. Different support options facilitated their work and strengthened employees’ sense of security and feeling of “being taken care of”. These included the presence of the security service and mobile teams dedicated to obtaining nasopharyngeal swabs from asymptomatic hospitalized patients to support the nurses. The employees appreciated the fact that they could be tested quickly in the hospital’s testing facility if they were symptomatic, that they had free parking near the hospital and overnight accommodation for cross-border commuters. HCWs particularly appreciated personal support from superiors including time for discussion of difficult situations, such as infections among one’s team members, family members anxiety’ and sick relatives who needed care.

## Discussion

We identified the following institutional actions, strategies, and policies as being related to HCWs’ safety perception. *First*, timely provision of clear and transparent information to all employees. As an example, transparent communication regarding the availability of PPE strengthened the feeling of safety, even if the information communicated was that specific items of PPE were scarce. Clear and regular communication regarding the correct use of PPE can prevent misunderstandings and ambiguities. Furthermore, providing digital access to key data is of critical importance to the employees. *Second*, in-person information transfer is superior to digital resources when the goal is to strengthen the employees’ perception of safety. Yet, supplementary digital resources can help reach all HCWs easily and rapidly (especially those working shifts). *Third*, to achieve uniform implementation of IPC recommendations, sufficient time must be provided to allow for the adoption of revised instructions. Regular meetings and possibilities for exchange between clinics are important to avoid inconsistencies in the implementation of the guidelines and will allow uniform adjustments to be made across an institution. *Fourth*, support and appreciation from superiors and leaders enhance HCWs’ sense of security.

Our findings are in line with other reports. Early in the course of the pandemic, Liu et al. noticed that HCWs needed support and regular intensive training [3]. Piché-Renaud et al. found that correct and regular training on handling PPE was necessary even beyond the settings of public health emergencies such as a pandemic [20]. Further, Simms et al. found that HCWs’ perception of PPE availability rather than the communication of numbers of critical items available affected HCWs’ ability to perform work duties safely and was associated with long-term mental health consequences [21].

The qualitative nature of the study provides in-depth insight into the experience of a diverse group of HCWs during the pandemic. However, the generalization of the findings is limited due to the qualitative study approach. Validity can never be truly reached in qualitative research, as the interpretation of the findings is always influenced by the researcher’s position. As the coding was done by one researcher, we could not determine intercoder reliability. However, all material was double coded to check consistency as recommended [10]. The interviews were conducted by an early career research and medical doctorate candidate (SE) who was not known to the HCWs beforehand, thus ensuring relative neutrality and objectivity. Nevertheless, SE was perceived as an employee of the division of infectious diseases & hospital epidemiology, so that close and ongoing collaboration with this division and the project leader (STS) may have influenced the HCWs’ responses and the analysis of the findings. Recall bias could be present, as the interviews were conducted a few months after the initial phase of the pandemic in Switzerland.

### Conclusion

This study identifies institutional policies and actions, the most important of which being transparent communication, influencing HCWs’ safety perception during the first wave of the COVID-19 pandemic. Such knowledge reveals potential areas of action critical to improving preparedness and management in hospitals faced with an infectious disease threat.

## Supplementary Information

**Additional file 1: Table S1**. Characteristics of participants.

**Additional file 2: Table S2**. Interview Guide.

**Additional file 3: Table S3**: COREQ (Consolidated criteria for reporting qualitative studies): 32-item checklist.

## Data Availability

The dataset generated and analysed during the current study is not publicly available due to individual privacy possibly being compromised but is available from the corresponding author on reasonable request.

## Abbreviations

COVID-19: Coronavirus disease of 2019
HCWs: Healthcare workers
IPC: Infection prevention and control
PPE: Personal protective
SARS-CoV-2: Severe acute respiratory syndrome coronavirus 2

## References

[1] Bandyopadhyay S, Baticulon RE, Kadhum M, Alser M, Ojuka DK, Badereddin Y (2020). Infection and mortality of healthcare workers worldwide from COVID-19: a systematic review. BMJ Glob Health.

[2] Sun N, Wei L, Shi S, Jiao D, Song R, Ma L (2020). A qualitative study on the psychological experience of caregivers of COVID-19 patients. Am J Infect Control.

[3] Liu Q, Luo D, Haase JE, Guo Q, Wang XQ, Liu S (2020). The experiences of health-care providers during the COVID-19 crisis in China: a qualitative study. Lancet Glob Health.

[4] Weilenmann S, Ernst J, Petry H, Pfaltz MC, Sazpinar O, Gehrke S (2021). Health care workers' mental health during the first weeks of the SARS-CoV-2 pandemic in Switzerland—a cross-sectional study. Front Psychiatry.

[5] Marjanovic Z, Greenglass ER, Coffey S (2007). The relevance of psychosocial variables and working conditions in predicting nurses' coping strategies during the SARS crisis: an online questionnaire survey. Int J Nurs Stud.

[6] Tan R, Yu T, Luo K, Teng F, Liu Y, Luo J, et al. Experiences of clinical first-line nurses treating patients with COVID-19: a qualitative study. J Nurs Manag. 2020. 10.1111/jonm.13095 PMC7404505 32657465

[7] Bremer D, Härter M, Scherer M, von dem Knesebeck O, Koch-Gromus U. Auswirkungen der COVID-19-Pandemie auf die klinische Versorgung, Arbeitsprozesse und Mitarbeitenden in der Universitätsmedizin: Ergebnisse einer Interviewstudie am UKE. Das Gesundheitswesen. 2020. 10.1055/a-1226-6828 PMC7516358 32823355

[8] Sandelowski M (2010). What's in a name? Qualitative description revisited. Res Nurs Health.

[9] Sandelowski M (2000). Whatever happened to qualitative description?. Res Nurs Health.

[10] Schreier M (2012). Qualitative content analysis in practice.

[11] Tong A, Sainsbury P, Craig J (2007). Consolidated criteria for reporting qualitative research (COREQ): a 32-item checklist for interviews and focus groups. Int J Qual Health Care.

[12] Kuckartz U. Analyzing qualitative data with MAXQDA: text, audio, and video. In: Rädiker S, editor, 1st edn. Cham: Springer; 2019.

[13] Doyle S (2007). Member checking with older women: a framework for negotiating meaning. Health Care Women Int.

[14] Creswell JW (2009). Research design: qualitative, quantitative, and mixed methods approaches.

[15] Weiss RS (1995). Learning from strangers: the art and method of qualitative interview studies.

[16] Padgett DK (2016). Qualitative methods in social work research.

[17] Lincoln YS, Lincoln YS, Guba EG, Guba EG (1985). Naturalistic inquiry.

[18] Ely M, Anzul M, Freidman T, Garner D, McCormack-Steinmetz A (1991). Doing qualitative research: circles within circles.

[19] Birt L, Scott S, Cavers D, Campbell C, Walter F (2016). Member checking: a tool to enhance trustworthiness or merely a nod to validation?. Qual Health Res.

[20] Piché-Renaud PP, Groves HE, Kitano T, Arnold C, Thomas A, Streitenberger L, et al. Healthcare worker perception of a global outbreak of novel coronavirus (COVID-19) and personal protective equipment: survey of a pediatric tertiary-care hospital. Infect Control Hosp Epidemiol. 2020:1–7. 10.1017/ice.2020.415 PMC7468688 32782038

[21] Simms A, Fear NT, Greenberg N (2020). The impact of having inadequate safety equipment on mental health. Occup Med (Lond).

