# Supplementary material for: A qualitative study on safety perception among healthcare workers of a tertiary academic care center during the SARS-CoV-2 pandemic
Source: Antimicrob Resist Infect Control. 2022 Feb 8;11:30. doi: 10.1186/s13756-022-01068-0 (PMC8821840; doi:10.1186/s13756-022-01068-0)
Supplement: Supplementary file 1 — Additional file 1: Table S1. Characteristics of participants. [file 13756_2022_1068_MOESM1_ESM.docx]

*Additional Table 1*

**Characteristics of participants**

| *Nr.* | *Profession* | *Department* | *Position* |
| --- | --- | --- | --- |
| P1 | Physician | Department of Infectious Diseases and Hospital Hygiene | Senior |
| P2 | Physician | Department of Infectious Diseases and Hospital Hygiene | Senior |
| P3 | Physician | Department of Infectious Diseases and Hospital Hygiene | Non-Senior |
| P4 | Physician | Department of Infectious Diseases and Hospital Hygiene | Non-Senior |
| P5 | Physician | Cohort Ward | Senior |
| P6 | Physician | Department of Infectious Diseases and Hospital Hygiene | Senior |
| P7 | Physician | Women`s Clinic | Senior |
| P8 | Physician | Anaesthesiology | Senior |
| P9 | Physician | Cohort Ward | Non-Senior |
| P10 | Physician | Intensive Care Unit | Senior |
| P11 | Physician | Department of Infectious Diseases and Hospital Hygiene | Non-Senior |
| P12 | Physician | Intensive Care Unit | Non-Senior |
| P13 | Physician | Anaesthesiology | Non-Senior |
| P14 | Physician | Emergency Department | Senior |
| P15 | Physician | Emergency Department | Senior |
| P16 | Physician | Outpatient Clinic | Senior |
| P17 | Physician | Nephrology | Senior |
| P18 | Physician | Outpatient Clinic | Non-Senior |
| P19 | Physician | Emergency Department | Senior |
| N1 | Nurse | Cohort Ward | Senior |
| N2 | Nurse | Anaesthesiology | Non-Senior |
| N3 | Nurse | Cohort Ward | Senior |
| N4 | Nurse | Intensive Care Unit | Senior |
| N5 | Nurse | Cohort Ward | Non-Senior |
| N6 | Nurse | Women`s Clinic | Senior |
| N7 | Nurse | Cohort Ward | Non-Senior |
| N8 | Nurse | Anaesthesiology | Non-Senior |
| N9 | Nurse | Emergency Department | Non-Senior |
| N10 | Nurse | Department of Infectious Diseases and Hospital Hygiene | Non-Senior |
| N11 | Nurse | Women`s Clinic | Non-Senior |
| N12 | Nurse | Outpatient Clinic | Non-Senior |
| N13 | Nurse | Department of Infectious Diseases and Hospital Hygiene | Non-Senior |
| N14 | Nurse | Outpatient Clinic | Non-Senior |
| N15 | Nurse | Cohort Ward | Senior |
| N16 | Nurse | Emergency Department | Senior |
| N17 | Nurse | Intensive Care Unit | Non-Senior |
