# Supplementary material for: A qualitative study on safety perception among healthcare workers of a tertiary academic care center during the SARS-CoV-2 pandemic
Source: Antimicrob Resist Infect Control. 2022 Feb 8;11:30. doi: 10.1186/s13756-022-01068-0 (PMC8821840; doi:10.1186/s13756-022-01068-0)
Supplement: Supplementary file 2 — Additional file 2: Table S2. Interview Guide. [file 13756_2022_1068_MOESM2_ESM.docx]

*Additional Table 2*

**Interview Guide [translated from German to English]**

*Basic questions (related to all topics):*

- What positive and negative aspects can you think of about topic "X"? [What was good/bad?]

- Which aspects would you implement in the same way in the future and which differently?

- What wishes do you have for your department/area in relation to this topic?

*Opening questions*

- First of all, I would like you to describe your function at the USB in the past months and how long you have been working in this position.

- How long have you worked in contact with COVID-19 patients?

- Did you have any previous experience in isolation management and how long was it?

1. **Material** **availability**

[Masks (mouth-nose protection, FFP2), gloves, goggles, over-aprons, hand sanitizer, surface sanitizer, other materials?]

How did you perceive instructions regarding the correct protective equipment?

[Positive/negative, understanding of indication, material (which material should be used when), procedure (sequence, donning and doffing)].

2. **Infection Prevention and Control guidelines (COVID-19 manual)**

[Understanding, availability, completeness, currency/updates]

3. **Training options for the use of protective equipment.**

[Options: Pictorial instructions, videos, on site, walk in, experience with untrained staff (e.g. military, medical students, MA from other hospitals)]

4. **Safety**

[personal infection risk, theft, visitor management, security guards]

5. **Communication**

[internal, external, availability of information, interdisciplinary cooperation],

How did you perceive the collaboration with the Federal Office of Public Health or cantonal medical service, if you had one?

6. **Diagnostics and testing strategy**

[Turnaround time, access to results, marking in electronic database, transport of samples]

[Diagnostic algorithms, outpatient test center, inpatient test management, mobile smear teams]. Smear teams]

7. **Surveillance (Data Management)**

[Handling of data, overview of infected persons, hospitalized persons, etc.]

8. **Science/research**

[own research, implementation of new findings from research] 9.

9. **Preparation**

1. How well prepared did you feel for a pandemic in your work environment?
2. Did your area/department have a pandemic concept so far or do you know if one is now being planned?
   1. If so, how long has it been in place and could it be used?
   2. If not, what are the arguments against developing such a concept?

10. **Personal Lessons Learnt**

1. What lessons do you draw from the past months in relation to the IPC measures?
   [more general lessons learned]

11. Are there any other points you would like to add that I have not addressed?
