# Supplementary material for: A qualitative study on safety perception among healthcare workers of a tertiary academic care center during the SARS-CoV-2 pandemic
Source: Antimicrob Resist Infect Control. 2022 Feb 8;11:30. doi: 10.1186/s13756-022-01068-0 (PMC8821840; doi:10.1186/s13756-022-01068-0)
Supplement: Supplementary file 3 — Additional file 3: Table S3: COREQ (Consolidated criteria for reporting qualitative studies): 32-item checklist. [file 13756_2022_1068_MOESM3_ESM.docx]

*Additional Table 3*

**COREQ (Consolidated criteria for reporting qualitative studies): 32-item checklist.** (1)

| **Number & Item** | **Guide questions and description** | **Additional comments** | **Reported on page no. or**  **not applicable (N/A)** |
| --- | --- | --- | --- |
| **Domain 1: Research team and reﬂexivity** | | | |
| *Personal Characteristics* | | | |
| 1. Interviewer/  facilitator | Which author/s conducted the interview or focus group? | SE | Data collection, p. 6 |
| 2. Credentials | What were the researcher’s credentials? E.g. PhD, MD | cand. med. | N/A |
| 3. Occupation | What was their occupation at the time of the study? | medical student and doctoral candidate who was employed by the University Hospital Basel (USB) during the study period. | N/A |
| 4. Gender | Was the researcher male or female? | female | N/A |
| 5. Experience and training | What experience or training did the researcher have? | SE had no previous experience in qualitative research but deepened her knowledge by literature research and attended online courses to best understand and implement the methodology. | N/A |
| *Relationship with participants* | | | |
| 6. Relationship established | Was a relationship established prior to study commencement? | No. | N/A |
| 7. Participant knowledge of the interviewer | What did the participants know about the researcher? e.g. personal goals, reasons for doing the research | Since SE was not known to the participants, her role and function, as well as the goal and procedure of the interviews, were explained to the participants in the informed consent beforehand and declared again in the opening remarks before the interview began. | N/A |
| 8. Interviewer characteristics | What characteristics were reported about the interviewer/facilitator? e.g. Bias, assumptions, reasons and interests in the research topic | The participants were aware SE was a doctoral candidate and medical student interested in the “Lessons Learnt” of the acute first phase of the SARS-CoV-2 pandemic. | N/A |
| **Domain 2: study design** | | | |
| *Theoretical framework* | | | |
| 9. Methodological orientation and Theory | What methodological orientation was stated to underpin the study? e.g. grounded theory, discourse analysis, ethnography, phenomenology, content analysis | Data were analyzed using content analysis according to Schreier. | Data analysis, p. 7 |
| *Participant selection* | | | |
| 10. Sampling | How were participants selected? e.g. purposive, convenience, consecutive, snowball | The sampling was purposeful, and criterion based. | Participants, p. 5 |
| 11. Method of approach | How were participants approached? e.g. face-to-face, telephone, mail, email | Contact was made by email. | N/A |
| 12. Sample size | How many participants were in the study? | Out of 41 contacted employees, 36 participated. | Participants, p. 5 |
| 13. Non-participation | How many people refused to participate or dropped out? Reasons? | Five employees that were contacted did not participate for the following reasons: two did not respond within time for study inclusion, one was subsequently rejected because he had only started working at the hospital after the acute first phase of the pandemic, and two people forwarded the researcher to more suitable participants than themselves. No one dropped out once agreeing to participate. | N/A |
| *Setting* | | | |
| 14. Setting of data collection | Where was the data collected? e.g. home, clinic, workplace | The interviews were conducted either in the office of the employee, a meeting room in their department or in a private meeting room arranged by the interviewer on hospital campus. | N/A |
| 15. Presence of non-participants | Was anyone else present besides the participants and researchers? | Besides the researcher and participant, no one else was present during the interviews. | N/A |
| 16. Description of sample | What are the important characteristics of the sample? e.g. demographic data, date | Fully presented in Methods section | Participants, p. 5 |
| *Data collection* | | | |
| 17. Interview guide | Were questions, prompts, guides provided by the authors?  Was it pilot tested? | The interview guide is provided as supplementary material (translated from German to English). Two pilot tests were carried out. | Additional Table 2 |
| 18. Repeat interviews | Were repeat interviews carried out? If yes, how many? | No repeat interviews were carried out. | N/A |
| 19. Audio/visual recording | Did the research use audio or visual recording to collect the data? | The interviews were audio-recorded. | Data collection, p. 6 |
| 20. Field notes | Were ﬁeld notes made during and/or after the inter view or focus group? | SE took field notes during the interview and added to the interview protocol afterwards. These included noticeable points such as whether the participant came noticeably prepared with notes or emphasized on individual points particularly. | N/A |
| 21. Duration | What was the duration of the interviews or focus group? | On average, one interview lasted 30.05 minutes (between 14.25 min - 50.51 min). | Findings, p. 8 |
| 22. Data saturation | Was data saturation discussed? | Yes, data saturation was achieved. | N/A |
| 23. Transcripts returned | Were transcripts returned to participants for comment and/or correction? | The transcripts were not returned. | N/A |
| **Domain 3: analysis and ﬁndings** | | | |
| *Data analysis* | | | |
| 24. Number of data coders | How many data coders coded the data? | The interviewer SE coded the data and discussed her findings with other researchers in the project team. | Data analysis, p. 7 |
| 25. Description of the coding tree | Did authors provide a description of the coding tree? | No coding tree is provided. | N/A |
| 26. Derivation of themes | Were themes identiﬁed in advance or derived from the data? | The themes ermeged inductively from the text. | Data analysis, p. 7 |
| 27. Software | What software, if applicable, was used to manage the data? | MAXQDA | Data analysis, p. 7 |
| 28. Participant checking | Did participants provide feedback on the ﬁndings? | Yes, a member check was carried out. | Data analysis, p. 7 |
| *Reporting* | | | |
| 29. Quotations presented | Were participant quotations presented to illustrate the themes/ﬁndings? Was each quotation identiﬁed? e.g. participant number | Yes, participant quotations are presented to illustrate the themes and each quotation is identified by participant number. | Findings, p. 8 |
| 30. Data and ﬁndings consistent | Was there consistency between the data presented and the ﬁndings? | The results are based on the interview statements | Findings, p. 8 |
| 31. Clarity of major themes | Were major themes clearly presented in the ﬁndings? | Yes, major themes are clearly identified. | Findings, p. 8 |
| 32. Clarity of minor themes | Is there a description of diverse cases or discussion of minor themes? | Yes, minor themes are clearly identified. | Findings, p. 8 |

**References**

1. Tong A, Sainsbury P, Craig J. Consolidated criteria for reporting qualitative research (COREQ): a 32-item checklist for interviews and focus groups. International journal for quality in health care. 2007;19(6):349-57.
